# Supplementary material for: Detection of Fused Genes in Eukaryotic Genomes using Gene deFuser: Analysis of the Tetrahymena thermophila genome
Source: BMC Bioinformatics. 2011 Jul 11;12:279. doi: 10.1186/1471-2105-12-279 (PMC3143110; doi:10.1186/1471-2105-12-279)
Supplement: Additional file 1 — Results of Gene deFuser for the Tetrahymena thermophila genome. This zip file contains the raw results of the analysis of the Tetrahymena genome using Gene deFuser. To view the contents, unzip the file and open the Final_Tet.html file in the resulting folder. [file 1471-2105-12-279-S1.ZIP › Results/3701.m00213.html]

Gene deFuser -- Results of Job Final\_Tet

 


Gene deFuser

| Home | Retrieve Results | References | Help |
| --- | --- | --- | --- |

Back to Main Results of Job Final\_Tet

# Query Name: 3701.m00213

Candidate fusion gene

## Query Sequence:

MGKVFQFYEGKTVLLTGITGFLGKVIFEKFLRTLPMVKRIYVLIRSKKGSPVEERFKKVIHDSEIFERLRQEKGDNFFNYLFSKVVPIEGDLLKEGLGLSKQDYQTITQEANIVINCAASVDFNAKLEEAININVRGSLRMMELSKQCLQLENFVHVSTCYVNSDKRGWIEEDIYNTEQNARQLMDDLMKMPPAELERQTKTILGNYPNTYVFTKSAVERIIKAERPPNMTITIVRPSIIGAAVSEPCVGWVEGVTAASAVFLLSGIGMLKYIHANRNAIGDVVPVDVVSDQIIVTGALCANLRDLSVFNASTSSRNPMLWEVSERNTVQYWRANPPEKRLGSCEAQLIKDERHLRFKQIMRRIPALAYLKLSNFVGSENMKKNANRYLKIIYKAESVSATFSHFASNEWIFDSRKIEKMIDQLDEQEKNAFYLDVSGINWDNYILMFNWGMHRYVLNEKVEPPVSDKNDILKINNQAKYPYFSDINWALSSGKPFKPRSYEEYKSLLFNSSAVQDAISLVIEQQTKKQGFSQGLVENVNKEAHAIIKEMISNYKMSTLRMMAWFLHKVFKQIYEKVVIDDVNLKALANHNVKKDGPLIMIPTHRSYIDFLLVSYIFFAYKMQCPHIAAAEDFLNVTVVHHILRACGAFFTKRNQKENPLYKAIFYEYVQRLLMDDCMLEFFVEGTRSRSGKMLHPKFGIMGIVADAVFDSKIPDAKIVPITLNYDKVLEGDTFPYELLGEEKVKESLPRLIKAIKTLSMNFGRIHVNICEPMSIKQYLATNYPTADLTNRVQRKSIIEDLSYKIEYKLTDALVCMPTGIVSAIMLQSRKGITEDKLINKVHWVSQRIVERGGILAFTPDVSGSMIATRSSMNLLEGILLKTKKNVFELEVTSDTEYKNVLMLSYYRNQIVHLFILEAIVCVALTAFGPNVAFKEGVSLERIWEESSFLIELFEREYVLPNVPKTLEQFSKTLLAKMIELKVLKVNESGKICIVDENQVSFYNSLIYPLVESYWSVLLNFYTLSQSKTKFMPLKKFLQQAQWFTENMHDERIIQHHESCSLDTIKNAIQKYKHFKLIRTYTDADKSGAEENILLLVNEEKLNELETHIQKFLKKGFSQSGSVSDLAKRTLLSEYPILARL

### Significant Ortholog Group Hits and their Scores:

| N terminus | | C terminus | |
| --- | --- | --- | --- |
| [I] KOG1221 Acyl-CoA reductase | 33.9393939393939 | [I] KOG3730 Acyl-CoA:dihydroxyactetone-phosphate acyltransferase DHAPAT | 9.97776510402292 |

#### Graphs (click to enlarge):

|  |  |
| --- | --- |
| BLAST of Query Sequence | Location of Ortholog Group Hits |
|  |  |

Contact: Andre Cavalcanti\_\_\_\_\_Last Modified September 14, 2010
